# Creutzfeldt-Jacob Disease: a case report

**DOI:** 10.1186/1757-1626-1-146

**Published:** 2008-09-09

**Authors:** Eren Gozke, Nursel Erdal, Muge Unal

**Affiliations:** 1 Department of Neurology, FSM Teaching and Research Hospital, Istanbul, Turkey; 2 Fatih Sultan Mehmet Egitim ve Arastirma Hastanesi, E-5 üzeri, Bostanci, Istanbul, Turkey

## Abstract

**Introduction:**

Creutzfeldt-Jacob Disease is the most frequently seen type of prion diseases. Its clinical findings consist of predominantly progressive dementia with a rapid onset, myoclonus, and also cerebellar, pyramidal, extrapyramidal and visual signs. Definitive diagnosis is established with histological examination of brain biopsy or autopsy materials. Occurrence of periodical spikes in EEG, observation of cortical signal alterations during diffusion weighted (DW) MRI studies, and detection of protein 14-3-3 in cerebrospinal fluid (CSF) substantiate the diagnosis.

**Case presentation:**

Seventy year-old male patient referred with complaints of weakness and involuntary movements in left arm, changes in behavior, and forgetfulness. He also developed akinetic mutism after nearly three months. In EEG periodic triphasic waves were seen. Despite the absence of any apparent pathological finding in T2 and FLAIR MRI, excluding signs of atrophy, on DW MRI hyperintense signal changes in cortical regions (cortical ribboning) were observed. Protein 14-3-3 in CSF was detected.

**Conclusion:**

Patients who have progressive dementia and associated atypical features should be investigated especially with DW MRI. Cortical ribboning is a very useful diagnostic sign for CJD.

## Introduction

Creutzfeldt-Jacob Disease (CJD) is a rarely seen neurodegenerative disease among the infectious spongioform encephalopathies. It has four subtypes as sporadic, familial, iatrogenic and variant forms thought to be transmitted with ingestion of infected meat products [1]. Mean age at the onset is 60 years with a yearly incidence of approximately 1/1.000.000. Firstly in 1982, Prusiner hypothetically proposed prions as causative infectious agents of CJD. In fact, normally a prion (PrPC) is a glycoprotein found in normal cells of humans and animals. In humans prion protein gene is localized on the short arm of the chromosome 20. Methionine/valine polymorphism on codon 129 of this gene is found to be associated with CJD. Infective prion (PrPSc) is a posttranslational product resulting from defective folding of the normal prion. These abnormal prions accumulate in cells leading to the formation of vacuolar degeneration and some fibrillar structures; subsequently brain takes the form of a sponge resulting in death [2].

In this article a case with a probable sporadic CJD, in which diagnosis was established based on medical history, clinical presentation, findings of diffusion weighted (DW) MRI, EEG and CSF in accordance with clinical diagnostic criteria of World Health Organization (WHO) is presented.

## Case presentation

**Figure 1:**
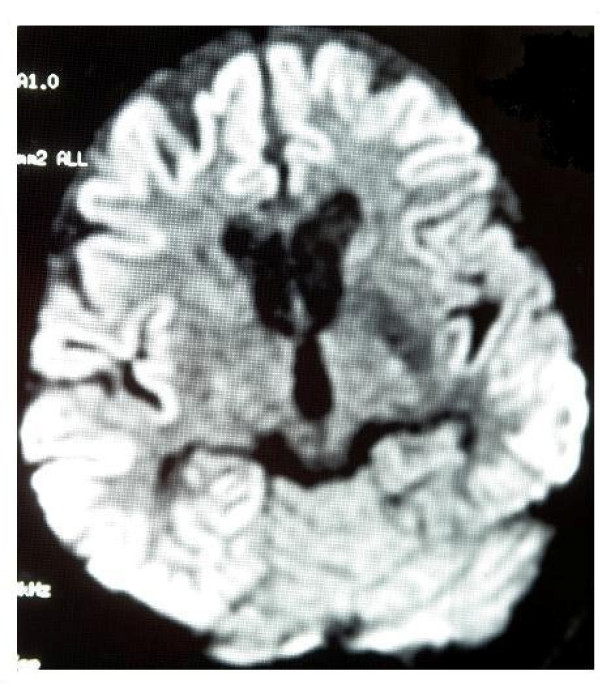
The cortical ribboning sign on diffusion MRI of the patient.

Three months before referral to our clinics, this 70 year-old patient experienced complaints such as difficulty in raising 3rd and 4th digits of his hands, insomnia, irritability, inability to find his way home. In cranial MRI bilateral cerebral cortical atrophy more prominent on the frontoparietal region was detected. The condition was evaluated as Alzheimer type dementia and cholinesterase inhibitors were initiated. To exclude the diagnosis of cervical radiculopathy and motor neuron disease, he underwent cervical MRI and EMG without detection of any specific finding. Two months after the onset of his complaints, visual hallucinations and tremor of the right hand were added. Cranial MRI was repeated without any detection of change. One month later neurologic examination revealed a mild degree of cognitive deficit, cerebellar signs in the right upper extremity and apraxia. The patient denied hospitalization for follow-up. Within ten days insomnia, irritability and agitation emerged, and he experienced delusions of being killed by their relatives, his speech became unintelligible and his gait instable. He was admitted to the hospital after a generalized tonic-clonic seizure. Neurological examination revealed drowsiness and disorientation. Facial asymmetry was absent, and extraocular movements were intact. Pupils were isochoric and at the midline with normal direct and indirect light reflexes. He was moving all his extremities in response to painful stimuli. Postural and action tremors and diffuse myoclonus emerging spontaneously and response to auditory or tactile stimuli were observed. Deep tendon reflexes were diminished, and planter reflexes were irrelevant bilaterally. Speech was extremely dysarthric and difficulty in swallowing was noted. Past medical history was unremarkable. Any abnormality besides lower TSH levels in laboratory tests could not be detected. From the 3^rd ^day of his hospitalization akinetic mutism developed. EEG showed 4–5 cps teta waves in background activity and also slow triphasic waves with higher amplitude on frontal regions were detected. In differential diagnosis Hashimoto encephalitis was contemplated secondary to lower levels of TSH. Laboratory findings of the patient were interpreted as subclinical hyperthyrodism. Marked cerebral atrophy in frontoparietal regions and several ischemic-gliotic foci were seen on cranial MRI, T2 weighted and FLAIR imaging, while hyperintense areas (cortical ribboning) all over the cortex was noted in DW MRI (Figure 1). Any increase in signal intensity was not detected in putamen and caudate nucleus. In CSF examination protein, glucose and electrolyte levels were within normal limits and no cell seen. However 14-3-3 protein was positive. Patient diagnosed as sporadic CJD and died within 4 months after the onset of his complaints.

## Discussion

CJD is a fatally progressive prion disease characterized with rapidly deteriorating dementia. Sporadic human prion diseases are seen in 85–90% of the cases. Of the remaining cases, 1–2% is the infectious form acquired from an established source with the prion disease, 5–15% is familial autosomal dominant type inherited secondary to the mutation of prion protein gene localized on chromosome 20 [1]. New variant CJD (nvCJD) has been reported since middle of 90's. The mean age at the onset of the disease is 29 years, and its mean survival is 16 months. Typical features of sporadic CJD could not be observed. The protein 14-3-3 could not be detected in CSF. This variant leads to cerebral prion plaques, contrary to spongioform alterations in the brain tissue [3,4].

Familial CJD is associated with a relatively early onset (40–50 years of age) and a prolonged course (22–24 months). Typical EEG findings are not seen, and genetic studies revealed nearly 20 genes mutations. Iatrogenic form can be occur secondary to duramater graft implantation, corneal transplantation, surgical operations performed with contaminated instruments, and hormonal therapies using hypophyseal growth hormones [1,5].

Symptoms of sporadic CJD can appear at 50–70 years of age. It constitutes 90% of all cases with CJD and it is characterized with rapidly progressive dementia and myoclonus. Personality changes accompany cerebellar and visual symptoms. Ataxia is more marked in advanced cases and most patients have myoclonus manifesting as a response to auditory and tactile stimuli. In late stages patient develops akinetic mutism and myoclonus can disappear. Eighty percent of the patients die from infection, cardiac and respiratory failure within the first year [1]. CSF protein levels rarely rise in CJD. Detection of a proteinase inhibitor, 14-3-3 protein released from damaged neurons into CSF fortifies the diagnosis [3,4]. Zerr et. al. found that 14-3-3 protein is 94% sensitive and 84% specific for the disease [3]. This protein can be detected in viral encephalitis, Hashimoto encephalitis, amyotrophic lateral sclerosis, and other types of dementia. Besides 14-3-3 protein, markers such as neuron specific enolase, amyloid beta, tau protein, astrocytic protein S 100 and neopterin are being investigated [4]. EEG does not reveal any pathological finding excluding nonspesific manifestations as normal or diffuse slowing, and frontal rhythmic delta activity. Periodic biphasic or triphasic, synchronized sharp wave complexes occuring during middle or late stages of disese are typical and found 90% of the patients. Steinhoff in his series of 150 cases reported 64% sensitivity and 91% specificity for EEG examinations. [5]. Zerr et al. found 66% sensitivity and 74% specificity [3]. In terminal stages of the disease where myoclonus are absent, typical EEG findings can not be elicited [3,5].

In sporadic CJD cerebral atrophy, increase in signal intensity in putamen, caudate nucleus and cerebral cortex can be detected in imaging studies. Increased signal intensity in the cortex is called ribboning. Shiga et al. revealed 92.3% sensitivity and 93% specificity for DW MRI in their patients with definitive (n = 9) and probable (n = 36) diagnoses of CJD [6]. Recent studies demonstrated that even in very early stages of the disease pathological findings can be detected with DW MR [6-9]. Definitive diagnosis of CJD requires neuropathological examinations. Spongiofrom alterations, astroglyosis and neuronal losses are detected in brain tissues obtained with biopsy or post-mortem sampling. Detection of PrPSc reactivity with immunohistochemical staining and demonstration of protease resistant PrPSc have diagnostic value [2].

## Conclusion

According to diagnostic criteria of World Health Organization (WHO) for the probable diagnosis of CDJ, the presence of at least one criterion among typical EEG findings and 14-3-3 positivity for CSF samples or at least 2 criteria among myoclonus, visual disturbances, cerebellar, pyramidal or extrapyramidal findings and akinetic mutism together with progressive dementia are required. [10]. Among these criteria pathological EEG findings and the presence of 14-3-3 protein in CSF samples were mentioned without considering MR findings. We though that DW MRI should be appropriately considered among diagnostic armamentarium, because it is a non-invasive screening tool with higher sensitivity and specificity than biopsies.

## Consent

Written informed consent was obtained from the patient for publication of this case report and accompanying images. A copy of the written consent is available for review by the Editor-in-Chief of this journal.

## Competing interests

The authors declare that they have no competing interests.

## Authors' contributions

EG analyzed and interpreted the patient data. NE and MU performed examination and clinical observation of the patient, and was a major contributor in writing the manuscript. All authors read and approved the final manuscript.
